# Carbolic Acid—Its Actions and Therapeutic Uses

**Published:** 1880-01

**Authors:** J. G. Westmoreland

**Affiliations:** Atlanta, Ga.


### CARBOLIC ACID—ITS ACTIONS AND THERAPEUTIC USES.

By J. G. WESTMORELAND, M.D., Atlanta, Ga.

Considering the short length of time this remedy has been in general use, it has proven more potent than any with which we are familiar. It seems but the other day, induced by the few and short notices made of it, that the writer made carbolic acid the subject of experiment. First, as a hæmostatic, causing the blood more readily to coagulate, its powerful, safe and useful virtue was thoroughly established. Menorrhagia and purpura hemorrhagica, by its use, vanished as dew before the rising sun. Where other styptics had been given for days without benefit, the bleeding was arrested and the hemorrhagic tendency subdued in a few hours. The increased plasticity of the blood, afforded by its use, is truly remarkable. Debilitating uterine hemorrhage setting in upon the ordinary menstrual flow, from a want of this property in the circulating fluid, is often arrested in two hours, and the sanguineous exudation from the whole alimentary and urinary mucous membranes, in purpura, has been made to cease in a day, by the use of four grains, every three or four hours.

In the above allusion to the dependence of hœmostatic effect of this remedy upon direct action had on the blood itself, increasing its placticity, we do not intend to make it differ in this respect from other styptics. Lead, chloride of iron, etc., arrest hemorrhage by the same kind of action, but are generally inferior to carbolic acid for the purpose. The old theory, ascribing the restraining influence to astringent impression upon the blood-vessels and other tissues concerned in the hemorrhagic condition, is preposterous. Three or four grains of acetate of lead thrown into the whole mass of the circulation, would hardly reach any one small blood-vessel in sufficient strength, to make any decided impression upon it or the surrounding cellular tissue. Even when applied, locally, to a part from which blood is being effused, the most decided benefit is derived from the prompt coagulation that is induced, thereby mechanically interfering with the further escape of blood, notwithstanding the most powerful constringing influence of which the remedy is capable, may be thus had. In this influence made upon the blood by carbolic acid and other styptics, when received into the circulation, we have not only the more ready coagulation, after leaving the vessels, but a thickening of the fluid, which lessens the tendency to escape through the tissues. So that by the same action upon the blood, we have the mechanical and vital restraints just mentioned.

Local action upon inflamed, and otherwise diseased, tissues, is that for which carbolic acid is more particularly celebrated. In various degrees of strength, from five per cent. solution in water to the undiluted acid, it is used for this purpose. In full strength, applied by a camel's hair brush to parts affected with diphtheretic inflammation, the remedy affords prompt relief; and when the inflammation does not extend beyond the point where it can be thus applied, there is fair prospect of complete relief, from local manifestation of the disease. To the mouth of a child six months old, the acid does not produce more pain than other collutories, and in an hour removes the pseudo membrane, leaving the surface comparatively pale. The inflammatory condition, is in fact, relieved for the time, and by re-application, as the redness or formation of the white coat again commences, the tendency to return will cease. Diluted with seventy-five or eighty per cent of water, it may be used as a gargle with great benefit to the fauces and pharynx, where the brush cannot be readily applied. The nares may be injected with a somewhat weaker solution, when they are also the subject of the inflammation.

Unfortunately, however, the disease often extends into the œsophagus and trachea beyond the reach of the brush, gargles or injections. The best that can be done with the remedy under these circumstances, is to cause the patient to swallow suitable doses in proper state of dilution, and inhale the vapor. Membraneous croup, which has effusion of lymph, at any rate, similar to diphtheretic formation, would seem to call for inhalation of this vapor early in the disease. The writer's experience has not afforded proof of this, but reasoning from analogy, the conclusion has been arrived at. A trial was made in a fatal case, but at a period too much advanced to afford a proper test.

In endo-metritis, notwithstanding the fear of stenosis expressed by gynæcologists, we have repeatedly injected full strength acid into the cervix and cavity of the uterus, without unpleasant effects and with great benefit to the patient. Destruction of tissue is not made, as some suppose, so as by cicatrix to produce contraction of the part.

The local alterative or catheretic effect is doubtless equal to that of nitric acid, but being less destructive to the tissues, is not likely to lead to stenosis of the cervix or adherence of the uterine walls. A mixture of the acid with iodine, acts well for this purpose, and those fearful of it, undiluted, will find five or ten per cent. in Lugol's solution of iodine a very useful application.

To ulcers, of suspected malignancy, upon the os uteri, the strong acid has proved effectual in one case, and is being tested now with unexpected favorable progess in a case giving pretty clear evidences of malignant character.

For this purpose, no dilution should be had, and the remedy should be applied twice a week. The spongy cauliflower appearance and disposition to hemorrhage from slight touch, is very soon relieved by the application, and without giving any decided pain to the patient.

The disinfectant property of carbolic acid, makes it peculiarly useful in the treatment of the ulcers alluded to. Nothing is more useful to destroy putrid odor.

Carbolic acid is notoriously famous as an antiseptic. As a dressing in wounds and in surgical operations it is now considered almost indispensable. While Professor Tindall ascribes its benefits to the destruction of and protection against bacteria and other microscopic animalculæ, others, among whom the writer desires to be recorded, attribute the benefit to the antiphlogestic result of its action. When applied to healthy or diseased structures they at once lose the color of blood. The healthy skin is made almost perfectly white in a moment. An inflamed surface is whitened and remains pale for a considerable time after the application. Without having the action of an ordinary astringent, the blood is driven more perfectly from the tissues acted on than by the most powerful of constringing agents. Vascular congestion or engorgement is thus not only relieved in the surface to which it is applied, but actually prevented from occurring in an injured part. Inflammatory action is thus arrested and prevented. The spray of ten per cent. carbolized water differs in degree only from full strength acid in its action. It is thought bacteria cannot live in it of this strength, and by keeping up the application of spray during the operation, and dressing the part operated on with carbolized material, this microscopic insect, the supposed origin of all putrefaction, is destroyed before its septic work has been done. Hence surgical injuries having early attention in this way are said to heal without suppuration or unpleasant odor.

A more rational explanation, however, seems to be that of preventing inflammation and the consequent disorganization, by lessening the amount of blood in the part, and by the remedy being continued, the first stages of the inflammatory process cannot occur. Not only will inflammation be prevented in the living subject, but decomposition or putrescence in the dead. Hence, for embalming the dead so as, for an indefinite time to prevent putrefaction entirely, nothing is more certain than the injection into the blood vessels the proper amount of carbolized water of suitable strength.

